# A large-scale retrospective study of the overall survival outcome in nasopharyngeal carcinoma with hypertension in Chinese population

**DOI:** 10.18632/oncotarget.17483

**Published:** 2017-04-27

**Authors:** Pei Yang, Hesham Elhalawani, Yingrui Shi, Ying Tang, Yaqian Han, Yu Zhao, Fan Lou, Hekun Jin

**Affiliations:** ^1^ Department of Head and Neck Radiation Oncology, Hunan Cancer Hospital, Changsha, Hunan, China; ^2^ Department of Radiation Oncology, The University of Texas MD Anderson Cancer Center, Houston, TX, USA; ^3^ Department of Chinese Traditional Medicine, The Jishou Chinese Traditional Medicine Hospital, Jishou, Hunan, China

**Keywords:** VEGF, NPC, hypertension, radiation theropy, hypoxia

## Abstract

**Background:**

It is known that hypertension is associated with high levels of vascular endothelial growth factor (VEGF) expression which is, in turn, highly connected to the prognosis of a wide array of cancers. The purpose of this study was to evaluate the relationship between hypertension and prognosis of nasopharyngeal carcinoma (NPC) with definitive radiotherapy in a Chinese population.

**Patients and Methods:**

We retrospectively reviewed 4493 patients with NPC who received definitive radiotherapy from 1995 to 2006, with a minimum follow-up of 5 years. Kaplan-Meier survival analysis and Cox proportional hazard model were utilized to determine the association between hypertension and overall survival (OS).

**Results:**

A total of 802 patients with NPC suffered from hypertension as compared to 3691 patients with no associated hypertension. Kaplan-Meier analysis revealed median overall survival of 101.1 and 110.0 months, respectively (*p*<0.05). In univariate survival analysis, patients with hypertension had worse OS (*p*<0.05) than non-hypertension patients. Patients with higher grade hypertension also had worse OS (*p*<0.05) compare to patients with grade 1 hypertension. In multivariate survival analysis, patients with hypertension had significantly worse OS (*p*<0.05) than non-hypertension patients, as well as M stage (*p*<0.001), after adjustment for related clinical confounding factors.

**Conclusion:**

Our findings provide evidence that hypertension is an independent factor and result in poorer survival outcomes in patients with NPC, the mechanism is still unclear, and it worth further research.

## INTRODUCTION

Epidemiological evidence shows that high blood pressure is associated with elevated the risk of many kinds of cancers [1-3]. Furthermore, Harding et al. found hypertension, both treated and untreated to be associated with an increased risk of cancer incidence and mortality *via* pooled collaborative analysis of a large retrospective cohort [4]. Correspondingly, several studies claimed that essential hypertension had increased serum vascular endothelial growth factor (VEGF) levels [5, 6]. The mechanism was demonstrated that hypertension could cause the microvascular damage and trigger the response of vascular repairing *via* upregulate the expression of VEGF in plasma [7].

Interestingly, VEGF also known as vascular permeability factor is an important angiogenic agent and endothelial specific mitogen, which has been implicated in the neovascularization and cell proliferation of a wide variety of tumors [8, 9]. Moreover, the results of the current study show that VEGF expression is significantly increased in patients with histologically advanced NPC, and those showing recurrence and cervical lymph node involvement [10]. Furthermore, high expression of VEGF is also active cancer-related mitogen and highly related to cancer prognosis [11-13].

These findings have led to the suggestion that hypertension might have a correlation with survival outcome of cancer. Nasopharyngeal carcinoma (NPC) is a common malignancy in Southeast Asia, especially in the southern coastal area of mainland China, Hong Kong, Macao and Taiwan [14]. Hence, we formulated a study based on the hypothesis that hypertension in patients with NPC may independently influence the survival outcomes of this peculiar disease in a Chinese population.

## RESULTS

Baseline characteristics of subjects are shown in Table 1. The entire sample included 4493 subjects, 2633 patients’ dead cause were NPC-related, and 1860 patients were censored including non-cancer related death and track lost. A total of 799 subjects (17.8%) were found to be hypertensive, and 3694 (82.2%) were not before the treatment. Hypertension was more frequently encountered in the cohort of NPC patients older than 46 years of age (*p* < 0.001), and male cohort (*p* < 0.05). The prevalence of smokers and alcohol consumers in the entire cohort was 2505 (55.7%) and 1498 (33.4%), respectively. The other clinical characteristics are well balanced.

**Table 1:** Baseline characteristics between hypertensive and non-hypertensive patients with NPC

Variables	Hypertensive	Non-Hypertensive	*p*
	***N* (%)**	***N* (%)**	
**Age**			0.000
<46	311 (15.2%)	1737 (84.8%)	
≥46	491 (20.1%)	1954 (79.9%)	
**Gender**			0.047
Male	632 (18.5%)	2787 (81.5%)	
Female	170 (15.8%)	904 (84.2%)	
**KPS**			0.712
≥70	784 (17.9%)	3600 (82.1%)	
<70	18 (16.5%)	91 (83.5%)	
**Smoking**			0.394
Yes	458 (18.3%)	2047 (81.7%)	
No	344 (17.3%)	1644 (82.7%)	
**Alcohol**			0.297
Yes	280 (18.7%)	1218 (81.3%)	
No	522 (17.4%)	2473 (82.6%)	
**T Stage**			0.126
T0-2	435 (17.1%)	2111 (82.9%)	
T3-4	367 (18.8%)	1580 (81.2%)	
**N Stage**			0.395
N0-1	427 (18.3%)	1904 (81.7%)	
N2-3	375 (17.3%)	1787 (82.7%)	
**M Stage**			0.183
M0	782 (18.0%)	3565 (82.2%)	
M1	20 (13.7%)	126 (86.3%)	
**Chemotherapy**			0.901
Yes	659 (17.9%)	3026 (82.1%)	
No	143 (17.7%)	665 (82.3%)	
**Pathology**			0.430
Nonkeratinizing	738 (17.7%)	3426 (82.3%)	
Other	64 (19.5%)	265 (80.5%)	

*The pathology types of keratinizing and undifferentiated were combined in the other

**Figure 1:**
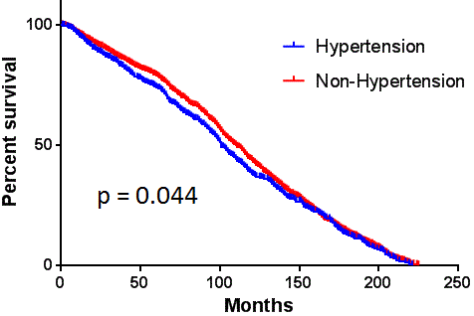
The comparison of overall survival between patients with and without hypertension

Kaplan-Meier analysis showed that patients with hypertension had significantly worse overall survival than that of subjects without hypertension, median survival month is 101.1 ± 2.9 and 110 ± 1.6, respectively (*p* < 0.05) (Figure 1), as well as the elder patient (*p* < 0.05).

**Figure 2:**
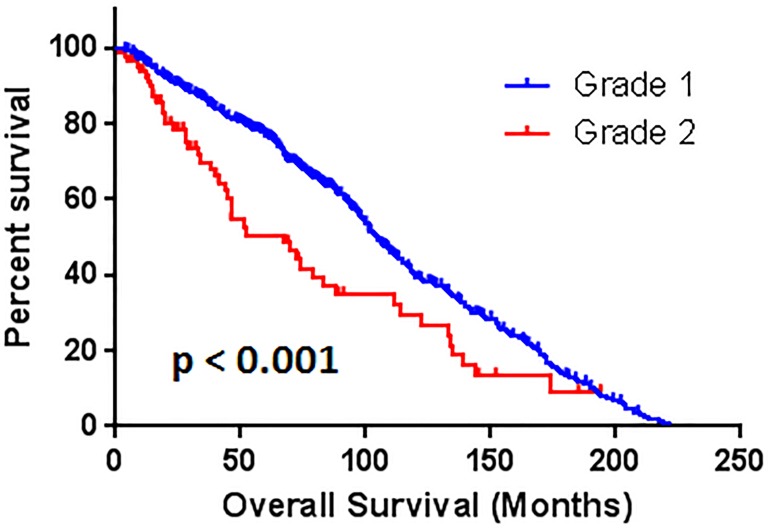
The comparison of overall survival between patients with grade 1 and grade 2

**Figure 3:**
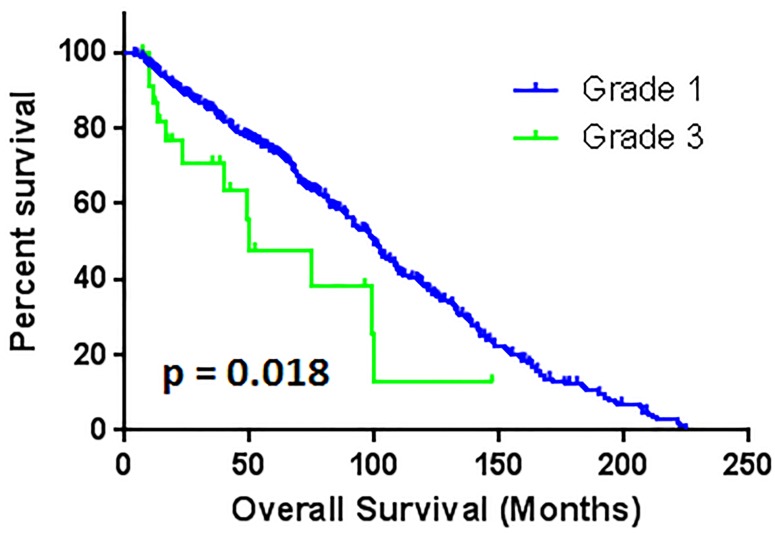
The comparison of overall survival between patients with grade 1 and grade 3

**Figure 4:**
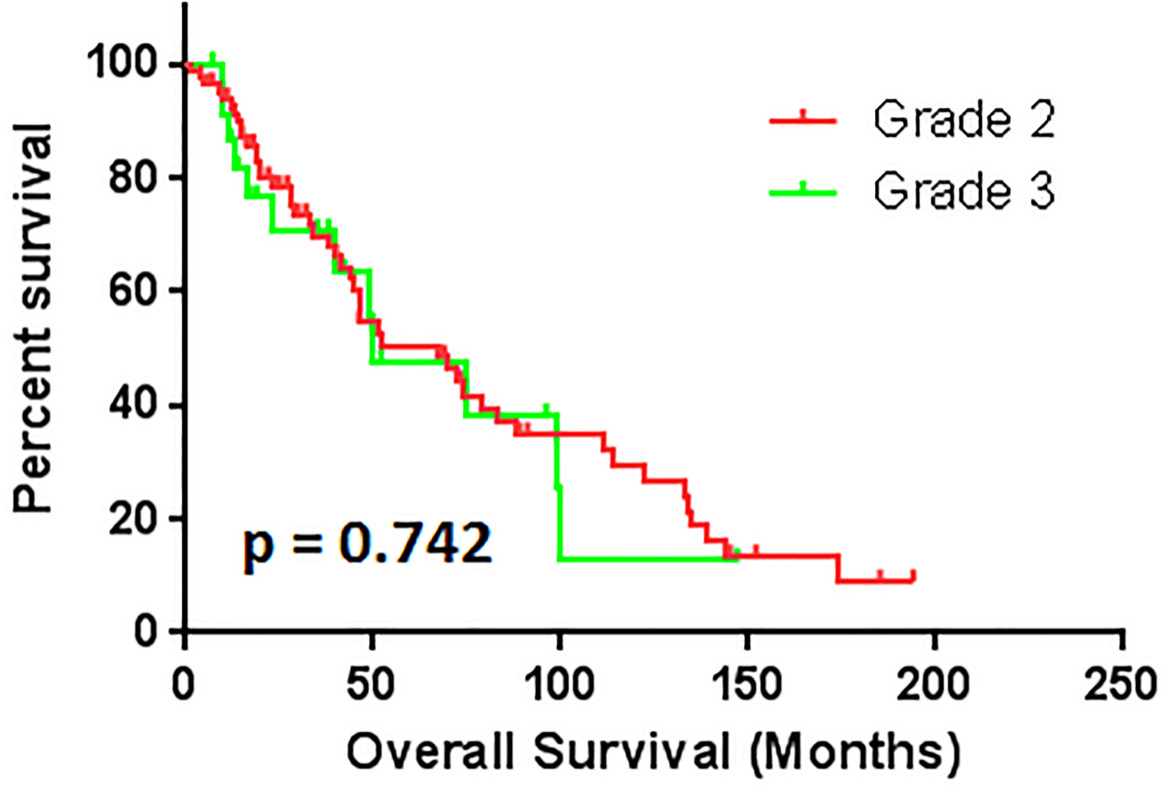
The comparison of overall survival between patients with grade 2 and grade 3

Correspondently, the median survival time are 104.9 ± 2.9, 67.5 ± 14.1, and 50.1 ± 19.7 months with grade 1, 2, and 3, respectively. The patients with grade 2 hypertension had worse overall survival outcome than grade 1 (*p* < 0.001) (Figure 2). The patients with grade 3 hypertension also had worse OS compare to patients with grade 1 (*p* < 0.05) (Figure 3). There are no statistical significant of overall survival between patients with grade 2 and grade 3 hypertension (*p* > 0.05). (Figure 4)

Univariate Cox proportional hazards regression analysis was performed to estimate risk factor for clinical outcomes. Results indicate that elder patients (*p* < 0.05), hypertension (*p* < 0.05), and positive metastatic disease (*p* < 0.001) were correlated with increased mortality in patients with NPC (Table 2).

**Table 2:** Uni-variate OS analysis of prognostic factors

Variables	OS	
	HR (95% CI)	*p*
**Age**		
<66	1.00	--
≥66	1.042 (0.956 – 1.137)	0.350
**Gender**		
Male	1.00	--
Female	0.979 (0.896-1.069)	0.636
**KPS**		
≥70	1.00	--
<70	1.085 (0.768 – 1.533)	0.644
**Smoking**		
Yes	1.00	--
No	1.035 (0.958 – 1.118)	0.381
**Alcohol**		
Yes	1.00	--
No	0.984 (0.907-1.068)	0.702
**Hypertension**		
None	1.00	--
Yes	1.107 (1.003 – 1.222)	**0.044**
**T stage**		
**T_0-2_**	1.00	--
**T_3-4_**	1.059 (0.979-1.146)	0.150
**N stage**		
**N_0-1_**	1.00	--
**N_2-3_**	0.966 (1.027-1.197)	0.378
**M stage**		
**M**_0_	1.00	--
**M**_1_	6.216 (4.989 – 7.745)	**<0.001**
**Pathology**		
Nonkeratinizing	1.00	--
Others	1.083 (0.930-1.263)	0.305
**Chemotherapy**		
Yes	1.00	--
No	0.936 (0.848-1.033)	0.188

Multivariate Cox proportional hazards regression analysis controlling potential confounders was conducted to evaluate association hypertension and clinical outcomes (Table 3). In this study, multiple analyses indicated that hypertension significantly increases the risk of mortality in patients with NPC after adjustment (RR = 1.110, 95.0% CI: 1.005-1.225 *P* = 0.039). (Table 4)

**Table 3:** Multi-variate OS analysis of all prognostic factors

Variables	OS	
	HR (95% CI)	*p*
**Age**		
<46	1.00	--
≥46	1.089 (1.008 – 1.176)	0.030
**Gender**		
Male	1.00	--
Female	0.997 (0.910-1.091)	0.997
**KPS**		
≥70	1.0	--
<70	1.133 (0.801 – 1.601)	0.480
**Smoking**		
Yes	1.00	--
No	1.034 (0.955 – 1.118)	0.408
**Alcohol**		
Yes	1.00	--
No	0.987 (0.908-1.073)	0.757
**Hypertension**		
None	1.00	--
Yes	1.111 (1.006 – 1.227)	**0.037**
**T stage**		
**T_0-2_**	1.00	--
**T_3-4_**	1.047 (0.968-1.133)	0.250
**N stage**		
**N_0-1_**	1.00	--
**N_2-3_**	0.970 (0.898-1.047)	0.430
**M stage**		
**M_0_**	1.00	--
**M_0_**	6.224 (4.993 – 7.758)	**<0.001**
**Pathology**		
Nonkeratinizing	1.00	--
Others	1.069 (0.917-1.246)	0.394
**Chemotherapy**		
Yes	1.00	--
No	0.926 (0.839-1.023)	0.129

Note: The pathology types of keratinizing and undifferentiated were combined in the other; all prognostic factors were analysis and adjusted using enter method in COX

**Table 4:** Multivariate survival analysis for nasopharyngeal carcinoma (final best model)

Variables	OS	
	HR (95% CI)	*p*
**Age**		
<46	1.00	-
≥46	1.087 (1.007 – 1.173)	**0.033**
**Hypertension**		
No	1.00	--
Yes	1.110 (1.005 – 1.225)	**0.039**
**M stage**		
**M_0_**	1.00	--
**M_0_**	6.257 (5.022 – 7.796)	**<0.001**

Note: All three significant factors in the model via back step method.

## DISCUSSION

To our knowledge, this is the first study to be conducted to evaluate the extent by which hypertension is associated within Chinese patients with NPC. The main finding was that NPC patients with hypertension have more inferior survival outcomes compared with those without hypertension. Unfortunately, no previous document has reported to relationships between NPC and elevated blood pressure or hypertension.

In general, upon hypertension-induced microvascular damage, a vast array of growth factors, cytokines, and other molecules are released, stimulating angiogenesis *via* VEGF, which is essential for the repair process and results in the high level of VEGF expression [7, 15]. Previous studies have also demonstrated that VEGF is increased in hypertensive patients and downregulated after control of blood pressure [5]. Correspondingly, several studies have claimed that essential hypertension patients also have increased serum VEGF levels [5, 6]. VEGF is a multifunctional glycoprotein which is mitogenic for endothelial cells and is the most important regulator of physiological or pathological angiogenesis, the process of new blood vessel growth from preexisting vessels, which is imperative to malignant tumor growth [16]. Many complex molecular pathways that govern tumor angiogenesis are logical targets for pharmacological manipulation given the important role they play in the growth and development of cancers. Along the same lines, VEGF was demonstrated as one of the most important factors in tumor angiogenesis by increasing blood vessel permeability, endothelial cell growth, proliferation, migration, and differentiation [17-19]. Moreover, overexpression of VEGF has been linked to tumor progression and poor prognosis in many tumor types [20-22].

**Figure 5:**
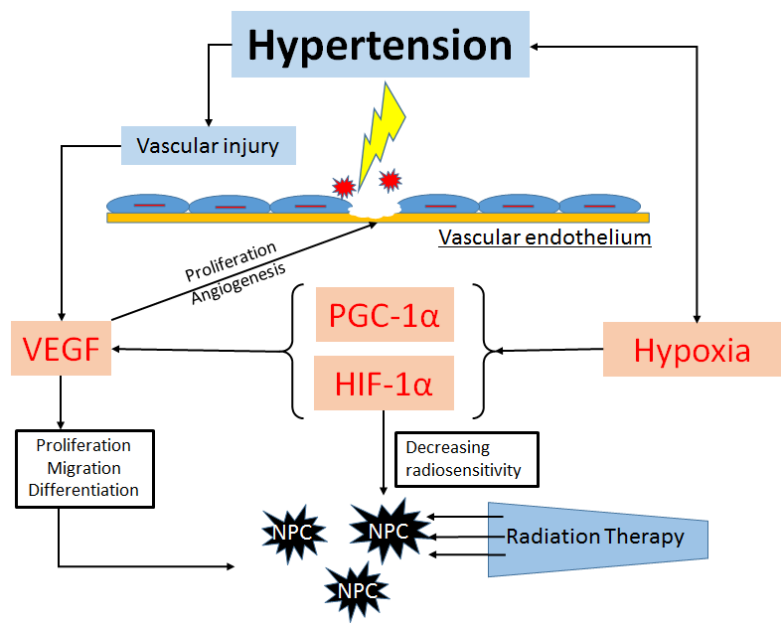
The possible mechanisms of hypertension affect the survival outcome in NPC patients

Furthermore, the chronic hypoxia causes increased systemic arterial pressure and massive activation of the sympathetic nervous system in healthy humans, that is to say, the patients with hypertension have the hypoxia inner environment exists [23]. Hypoxia itself was reported related to the worse clinical survival outcome [24]. The status of hypoxia upgrade the level of VEGF in response to a tumor microenvironment *via* the hypoxia-inducible transcription factor-1α (HIF-1α) and peroxisome proliferator-activated receptor-gamma coactivator (PGC-1α), Expression of VEGF in response to hypoxia is key to this process and has led to VEGF being defined as the prime hypoxia-inducible angiogenic factor [25, 26]. Moreover, hypoxia is also an important contributor to tumor radioresistance, the plasma level of HIF-1 decrease the radiosensitivity [27, 28]. However, radiotherapy is the major treatment in NPC patients; low radiosensitivity is strongly associated to the worse prognostic in head and neck cancers [29, 30]. Several studies also proved the high expression of VEGF-related to the poor survival prognostic in nasopharyngeal carcinoma cancer patients [11-13]. Hence, these connections could be the main reason that NPC patients with high-grade hypertension had worse survival outcome. (Figure 5)

In our findings, elder patients (age ≥ 46 years) had worse OS (*p* < 0.05), we figure the reason was that the percentage of hypertension distributed higher in the cohort of elder patients. This result is also consistent with several findings [31-33]. Unfortunately, all the researchers failed on taking hypertension as one of the potential factors.

However, there are several limitations in this study. First, the examination on the level of VEGF/HIF-1 in plasma of all patients was not available. Secondly, some studies indicate that anti-hypertension medications, such as beta-blockers, might have the potential effect of reducing the level of VEGF in plasma [34]. Unfortunately, we were unable to retrieve the drug usage in this cohort of patients. Moreover, most of NPC patients have the high expression of Epstein-Barr virus (EBV), and the correlation between VEGF expression and EBV status was explored, where EBV was suggested to have a role in angiogenesis and subsequent disease progression *via* VEGF upregulation, this mechanism could result in an interactive reinforcement in NPC patients. Unfortunately, the exam of EBV was not performed while this cohort of patients received radiotherapy from 1995 to 2006 [35].

Base on the mechanism above and the limitation of this retrospective study, our group have already been proceeding a relative large-scale prospective research on it.

In conclusion, our findings provide evidence that hypertension is associated with overall survival outcome of NPC patients, along with the positive metastatic disease. We postulate that the possible mechanism is the higher plasma VEGF level in the patients with hypertension. Although there are several limitations in this study, this finding still worth further research.

## MATERIALS AND METHODS

### Study population

We scanned our medical records of Chinese patients with NPC who were treated with definitive chemoradiotherapy from 1995 to 2006 at Hunan Cancer Hospital. Retrospectively recruited 4493 consecutive Chinese patients. The inclusion criteria were as follows: (a): histopathologically confirmed NPC, (b): receipt of definitive radiotherapy with or without concurrent chemotherapy. (c): no other primary cancer or a history of another cancer. Patients were excluded from the study to eliminate potential confounding factors which may influence the clinical outcome of NPC. Exclusion criteria included the following: 1) history or findings of significant valvular heart disease (i.e. more severe than mild valvular insufficiency or stenosis), hyperthyroidism or hypothyroidism or cardiomyopathy (dilated or hypertrophic); 2) atrial fibrillation; 3) pregnancy; and/or 4) a major systemic illness such as Systemic lupus erythematosus. A written consent was obtained from all patients before data accrual. Moreover, this study was approved by the Ethics Committee of the Hunan Cancer Hospital, Changsha, China.

### Measurement and definition

This patient database contained detailed demographic data, the patient’s status (smoking index, alcohol consumption measurements, etc.), comprehensive tumor details, clinical stage, histological subtype, radiotherapy (RT) data, chemotherapy data, treatment outcomes, and mortality data. The disease was restaged according to the seventh edition of the American Joint Committee on Cancer (AJCC) staging system [36], and the pathological tumor types were determined according to the World Health Organization (WHO) NPC classification [37]. The diagnosis of hypertension was derived from the medical records of every patient. We evaluated and categorized the patient`s hypertension grade with the average blood pressure based on the weekly result of blood pressure during the whole treatment in our hospital. The definition of hypertension was according to American Heart Association (AHA) guidelines [38]. All patients underwent definitive RT 5 days per week by using traditional conformal radiotherapy technology.

### Clinical endpoints

At a median follow-up of 80 weeks, all included patients were further contacted by telephone or mail to screen for new death events. The data set was completed by information obtained from relatives, attending physicians and hospital records. In this study, our clinical endpoints included both NPC-related deaths, as well as other non-cancer-related deaths, were also documented.

### Statistics analysis

Differences in the distribution of baseline characteristics between individuals with censor and outcome groups were examined using Chi-square test for categorical variables and Student’s *T*-test for continuous variables. Descriptive statistics are presented as percentages. All *P*-values are two-sided, and values of < 0.05 were considered to be statistically significant. For clean interpretation and summarization of results, Kaplan-Meier time-to-event analyses were used for clinical outcomes, with log-rank tests used for differences between previous study-group assignments. Cox proportional hazards regression models adjusted for potential confounders were used to study the relation between relative risk of death and hypertension severity at baseline. First, univariate Cox regression analyses were carried out to examine the association between each potential confounder and clinical outcomes. Potential confounders for outcomes included age, gender, smoking index, alcohol consumption, T, N and M categories, NPC WHO pathological subtypes, and hypertension grade of severity. Secondly, we fitted separate univariable Cox regression models to evaluate the influence of each covariate in the strength of association between hypertension severity and clinical outcomes. The adjusted hazard ratio of the results among NPC patients with hypertension compared with those without hypertension was the basic model from which the effect of each covariate was estimated. Estimates derived from Cox regressions are presented as hazard ratios and 95% confidence intervals (CI). Statistical analyses were performed using SPSS version 23.0

## Abbreviations

VEGF: vascular endothelial growth factor
NPC: Nasopharyngeal carcinoma
RT: radiotherapy
OS: overall survival
HIF-1α: hypoxia-inducible transcription factor-1α
PGC-1α: peroxisome proliferator-activated receptor-gamma coactivator
EBV: Epstein-Barr virus
AJCC: American Joint Committee on Cancer
WHO: World Health Organization
AHA: American Heart Association
